# Designing Ultra‐Narrow‐Band Red Phosphor via Oxygen Vacancy Engineering for Transparent Display Application

**DOI:** 10.1002/advs.202416761

**Published:** 2025-02-17

**Authors:** Wei Wang, Yi Wei, Hang Yang, Jinxuan Sun, Fuyan Su, Hanrui Liao, Hua Zou, Mingrui Li, Guogang Li

**Affiliations:** ^1^ College of Physics and Optoelectronic Engineering Hainan University 58 Renmin Avenue Haikou 570228 P. R. China; ^2^ Faculty of Materials Science and Chemistry China University of Geosciences 388 Lumo Road Wuhan 430074 P. R. China; ^3^ Department of Chemistry Southern University of Science and Technology Shenzhen Guangdong 518055 P. R. China

**Keywords:** anti‐thermal quenching, oxygen vacancy, red emission, transparent display, ultra‐narrow‐band

## Abstract

Narrow‐band red phosphors have been crucial in enabling energy‐efficient and wide color gamut display technologies. Developing novel red phosphors with narrower FWHM and suitable positions is still an urgent demand. Herein, a nanorod‐shaped Nb_2_O_5_:Pr^3+^ phosphor, featuring a single ultra‐narrow‐band red emission at 612 nm with FWHM of only 19 nm, is reported. The single narrow‐band is associated with oxygen vacancies, which can influence the electron–hole recombination energy. Besides, the intensity of PL spectra presents anti‐thermal properties and achieves an unexpected 12.5‐fold enhancement from 80 to 280 K. Detailed structural analyses, optical measurements, and DFT calculation are used to investigate abnormal photophysical processes. It canbe found that *V_O1_* has the lowest *E_form_* of 0.70 eV and the electron localization area around the Pr atom enlarges and presents the biggest distortion as *V_O1_* appears. The energy transfer from oxygen vacancies to the luminescent center accounts for the large enhancement. At last, the fabricated transparent display screen presents a transparency of 50% and high color purity (98%), and the LED device shows a large color gamut. These findings advance the understanding of the relationship between luminescent properties and oxygen vacancies, inspiring more design of narrow‐band red phoshors for display applications.

## Graphical abstract

A nanorod‐shaped Nb_2_O_5_:Pr^3+^ phosphor, featuring a single ultra‐narrow‐band red emission at 612 nm with FWHM of only 19 nm, is reported. The PL intensity presents anti‐thermal properties and achieves an unexpected 12.5‐fold enhancement from 80 to 280 K, the related relationship between luminescent properties and oxygen vacancies is systematically investigated.

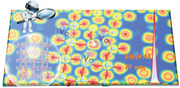

## 1 Introduction

Narrow‐band luminescent materials have evolved from being primarily used in high‐power lighting and liquid‐crystal display (LCD) applications to virtual reality (VR) and transparent display (TD) technologies, the push to develop narrow‐band inorganic phosphors, especially in the red region has been continuously received attention.^[^ ^1^ ^]^ The next‐generation high‐performance light‐emitting diodes (LED) for these devices need to meet these characteristics of high brightness, high resolution, and wide color gamut.^[^ ^2^ ^]^ The full width at half maxima (FWHM) and emission position (*λ*_em_) of red phosphors determine the color gamut of displays and the energy efficiency of LED.^[^ ^3^ ^]^ On the one hand, the human eye is less sensitive to the emission spectra that extend into the near‐infrared region (NIR), thus these parts of photons are lost and lower the efficiency of LED devices.^[^ ^4^ ^]^ Accordingly, using red phosphors with FWHM of 30 nm can reduce invalid emissions into the NIR and increase device efficacy by 22%.^[^ ^5^ ^]^ Therefore, setting a long‐term target that red phosphors used in LED should have FWHM below 30 nm helps to reduce energy consumption. On the other hand, the narrower FWHM means the higher color purity. For example, *β*‐SiAlON:Eu^2+^ (*λ*_em_ = 540 nm, FWHM = 55 nm) and K_2_SiF_6_:Mn^4+^ (*λ*_em_ = 630 nm, sharp emission) are widely used for display applications due to their narrow‐band emission.^[^ ^6^ ^]^ The fabricated device could achieve 110 lm W^−1^ and the calculated color gamut reached 96% of the National Television System Committee (NTSC) standard in CIE 1931.^[^ ^7^ ^]^ However, the fluoride red phosphor suffers from environmental pollution and poor chemical stability problems, which limits its wide application.^[^ ^8^ ^]^ Therefore, novel narrow‐band phosphors especially in the red region with excellent properties are still in huge demand for future electronic devices.

Actually, it is worth noting that achieving FWHM ≤ 30 nm is a really difficult task for inorganic phosphors. Until now, only a small number of single narrow‐band red phosphors have been reported that could fulfill the high requirements of practical applications.^[^ ^1^, ^9^ ^]^ The commercial red phosphors reported in the early days mainly include Eu^2+^ doped nitrides, such as CaSiAlN_3_:Eu^2+^ (*λ*_em_ = 640 nm, FWHM = 86 nm) and Sr_2_Si_5_N_8_:Eu^2+^ (*λ*_em_ = 625 nm, FWHM = 101 nm).^[^ ^10^ ^]^ The broad FWHM causes energy loss and is difficult to apply in the display field due to the low color purity. Then, researchers focus on the UCr_4_C_4_‐type phosphors because the highly symmetric site and condensed framework help to achieve narrow‐band emission.^[^ ^11^ ^]^ The classic red UCr_4_C_4_‐type phosphors, such as Sr[LiAl_3_N_4_]:Eu^2+^ (*λ*_em_ = 650 nm, FWHM = 50 nm), Sr[Mg_3_SiN_4_]:Eu^2+^ (*λ*_em_ = 615 nm, FWHM = 43 nm), and Sr[Li_2_Al_2_O_2_N_2_]:Eu^2+^ (*λ*_em_ = 614 nm, FWHM = 48 nm) present narrow band emission.^[^ ^1^, ^9^, ^12^ ^]^ However, these nitride phosphors are synthesized in the oxygen‐/waterfree glovebox and the process is harsh and complex, the FWHM exceeds 30 nm.^[^ ^13^ ^]^ Quantum dots (CsPbI_3_) and Mn^4+^‐doped fluorides (K_2_SiF_6_:Mn^4+^) are two other important red phosphors.^[^ ^14^ ^]^ Although FWHM of the two types is below 30 nm, the former suffers from toxicity and poor stability, the latter suffers from long decay times. Compared to the above, oxide phosphors have characteristics of simple synthesis, low cost, excellent chemical stability, and nontoxicity.^[^ ^15^ ^]^ Most red oxide phosphors are based on the Eu^3+^ or Mn^4+^ activators with multiple line emissions, there are few reports on single red narrow‐band emission and new methods need to be developed to accelerate the discovery of such oxide phosphors.

Inspired by the tool of oxygen vacancy engineering, we achieve a single ultra‐narrow‐band (19 nm) in a nanorod‐shaped Nb_2_O_5_:Pr^3+^ system. Pr^3+^ ions undergo 4*f*↔4*f* parity forbidden transitions, but energy could only transfer from the ^1^D_2_ level to the ground state (GS). Structural analysis and morphology study confirm the stable and pure phase, optical measurements indicate that Nb_2_O_5_:Pr^3+^ phosphor exhibits a wide excitation area and can match well with n‐UV LED chips. Unexpectedly, the intensity of PL spectra presents an outstanding 12.5‐fold enhancement from 80 to 280 K because of energy transfer from vacancies to the luminescent center. Density functional theory (DFT) calculation is conducted to analyze vacancy types and electron properties of Nb_2_O_5_:*x*Pr^3+^ system. Electron localization function (ELF) results indicate that the existence of *V_O1_* makes the electron localization area around the Pr atom enlarged and distorted. We investigate the underlying relationship between luminescent properties and oxygen vacancies, providing ideas for designing novel narrow‐band red oxide phosphors.

## 2 Results and Discussion

**Figure 1:**
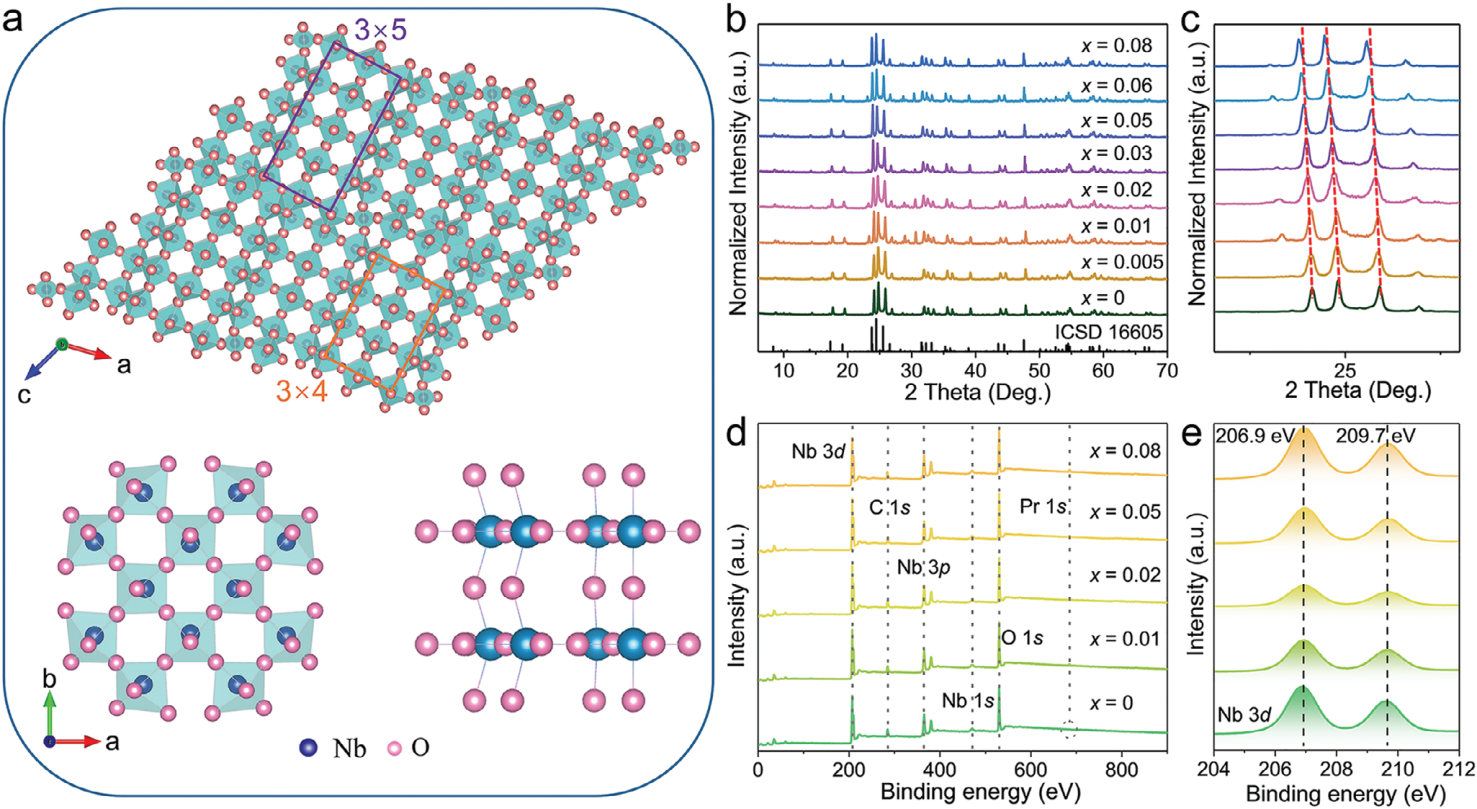
a) Crystal structure of Nb_2_O_5_ viewed along *b*‐axis direction (top) and *c*‐axis direction (bottom). b) PXRD patterns and c) selected diffraction peaks near 25° of Nb_2_O_5_:*x*Pr^3+^ (0 ≤ *x* ≤ 0.08) phosphors. The standard card (Nb_2_O_5_: ICSD‐16605) is used as a reference. d) XPS survey spectra of Nb_2_O_5_:*x*Pr^3+^ (*x* = 0, 0.01, 0.02, 0.05, 0.08). e) High‐resolution XPS spectra of Nb 3*d*.

A series of Nb_2_O_5_:*x*Pr^3+^ (0 ≤ *x* ≤ 0.08) samples were obtained by a traditional high‐temperature solid‐state method (see details in Experimental Section). **Figure** **1**a presents the crystal structure of the pristine Nb_2_O_5_ host according to Rietveld refinement results. The Nb_2_O_5_ structure crystallizes in an monoclinic cell with space group *P2*, the lattice constants (Table S1, Supporting Information) are *a* = 21.1682(4) Å, *b* = 3.8214(7) Å, *c* = 19.3531(3) Å, *α* = *γ* = 90°, *β* = 119.8254(1)°, and *V* = 1358.15(4) Å^3^, which is similar to the early reported results.^[^ ^16^ ^]^ As shown in Figure 1a, Nb_2_O_5_ lattice is constructed by 3 × 5 and 3 × 4 octahedral matrices, each matrix is connected with NbO_6_ octahedra by sharing the common oxygen atoms. Previous studies showed that this phase is a particularly stable type of Nb_2_O_5_, which perhaps becomes a good choice to accommodate rare earth ions as luminescent centers.^[^ ^17^ ^]^ Considering niobium cation is coordinated by six O atoms and forms the sole octahedra in the lattice, Pr^3+^ is expected to preferentially enter Nb site and form PrO_6_ octahedra. The powder X‐ray diffraction (PXRD) patterns of Nb_2_O_5_:*x*Pr^3+^ (0 ≤ *x* ≤ 0.08) samples in Figure 1b are in good consistency with the standard card (ICSD‐16605), confirming the formation of pure phase after introducing Pr^3+^ ions. Some characteristic peaks such as 2*θ* = 8.4°, 17.3°, 19.2°, 23.7°, 24.5°, 26.6°, 31.5°, 32.3°, 33.1°, 43.6°, and 47.5°, could be indexed as (‐201), (‐401), (10‐4), (110), (‐2‐11), (‐3‐11), (‐5‐12), (‐5‐11), (‐215), (315), and (020) planes of the reference. It could be observed that diffraction peaks near 25° (Figure 1c) shift to a smaller angle due to the ionic radius of Pr^3+^ (*r* = 1.13 Å, CN = 6, *r* is ionic radius, CN is coordination number) is larger than that of Nb^5+^ (*r* = 0.64 Å, CN = 6) according to Bragg's Law.^[^ ^18^ ^]^ The result indicates the successful incorporation of Pr^3+^ ions into Nb_2_O_5_ host. Figure S1 and Table S1 (Supporting Information) present the XRD Rietveld refinement results (conducted by GSAS software) of Nb_2_O_5_ host and the representative Nb_2_O_5_:0.01Pr^3+^ sample. The gained reliability factors and the absence of a secondary phase further confirm the pure phase. Compared to the pristine Nb_2_O_5_ host (*V* = 1358.15(4) Å^3^), the larger unit cell volume of Nb_2_O_5_:0.01Pr^3+^ (*V* = 1360.45(1) Å^3^) intuitively indicates that Pr^3+^ ions successfully enter the Nb sites. X‐ray photoelectron spectroscopy (XPS) spectra and fitting results in Figure 1d, e further prove the composition and valence states of Nb_2_O_5_:*x*Pr^3+^ phosphors, the C *1s* peak is used to calibrate the spectra. The principal XPS peaks of survey spectra are attributed to Nb *1s*, Nb *3p*, Nb *3d*, and O *1s* for Nb_2_O_5_:*x*Pr^3+^ (*x* = 0, 0.01, 0.02, 0.05, 0.08) phosphors, and Pr *1s* signal is also observed except for *x* = 0. The detailed high‐resolution (HR) XPS spectra of Nb *3d* are shown in Figure 1e, the observed signals at 206.9 and 209.7 eV are assigned to Nb *2d*_3/2_ and Nb *2d*_1/2_, respectively.^[^ ^19^ ^]^ In Figure S2 (Supporting Information), the characteristic Raman peaks of Nb_2_O_5_:*x*Pr^3+^ (*x* = 0, 0.01, 0.02, 0.05, 0.08) phosphors at 670 cm^−1^ are correlated with Nb‐O‐Nb bridging bonds of distorted NbO_6_ octahedra. Besides, the low‐frequency Raman peaks at 264 cm^−1^ are related to angle deformations of Nb‐O‐Nb bounds.^[^ ^20^ ^]^ These results confirm the composition and purity of the obtained crystalline phase.

**Figure 2:**
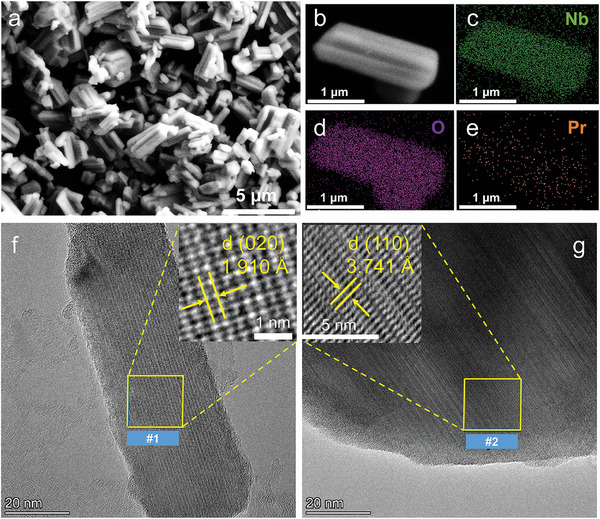
a) SEM image of Nb_2_O_5_:0.01Pr^3+^ sample. b) SEM image of a single rod and the corresponding mapping images of elements c) Nb, d) O, and e) Pr. HR‐TEM images and calculated lattice fringes of selected high‐resolution areas f) #1 and g) #2, respectively.

Scanning electron microscopy (SEM) is used to study the morphology and chemical composition of the selected Nb_2_O_5_:0.01Pr^3+^ phosphor. **Figure** **2**a reveals a large amount of relatively smooth rod‐like structure. Although some nanorods have gathered into clusters because of high‐temperature solid‐state reactions, the nanorods are geometrically uniform without impurities of other shapes.^[^ ^17^, ^20^ ^]^ The length of Nb_2_O_5_:Pr^3+^ nanorods is ≈2–5 µm, and we select a single nanorod (Figure 2b) for energy‐dispersive X‐ray spectroscopy (EDS) measurement. The elements Nb, O, and Pr are homogeneously distributed in the area (Figure 2c–e), EDX map of Pr is not as clear as Nb and O due to the concentration of doped Pr^3+^ is only 0.01. The Nb_2_O_5_:0.01Pr^3+^ phosphor is further studied by the corresponding high‐resolution transmission electron microscopy (HR‐TEM). As shown in Figure 2f, rod‐like structure and clear lattice fringes could be observed, indicating the high crystallinity of as‐prepared phosphor particles. The measured interplanar distances of 1.910 Å (Figure 2f) and 3.741 Å (Figure 2e) corresponding to (020) and (110) crystal planes are in agreement with the monoclinic cell of refined Nb_2_O_5_ structure. We select two other areas in Figure S3 (Supporting Information) to confirm the phase, the measured lattice d‐spacings of 3.739 Å (#3) is also indexed as (110) plane, the d‐spacings of 2.560 Å (#4) could be indexed as (‐802) crystal planes of rod‐like Nb_2_O_5_ structure. These results are well consistent with PXRD data and Rietveld refinement results in Figure 1.

**Figure 3:**
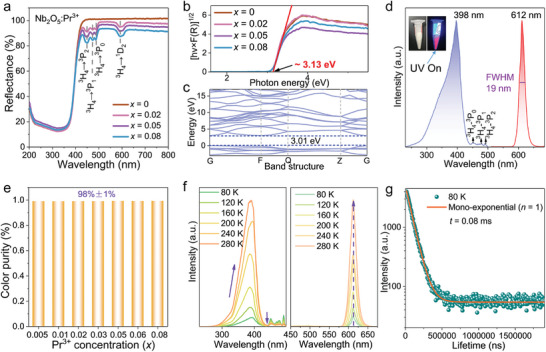
a) DR spectra and b) extrapolation of band gap energy of Nb_2_O_5_:*x*Pr^3+^ (*x* = 0, 0.02, 0.05, 0.08) samples. c) Band structure of Nb_2_O_5_ host calculated with DFT method. d) PLE and PL spectra of the representative Nb_2_O_5_:0.01Pr^3+^ phosphor. e) Color purity of Nb_2_O_5_:*x*Pr^3+^ (0.005 ≤ *x* ≤ 0.08). f) The temperature‐dependent PLE (left) and PL (right) spectra from 80 to 280 K of Nb_2_O_5_:0.01Pr^3+^ phosphor. g) PL decay curves and fitting results of Nb_2_O_5_:0.01Pr^3+^ at 80 K.

Diffuse reflectance (DR) spectra of Nb_2_O_5_:*x*Pr^3+^ (*x* = 0, 0.02, 0.05, 0.08) phosphors in **Figure** **3**a are recorded at room temperature. The range covering from 240 to 400 nm is atributed to the absorption of Nb_2_O_5_ host, so this absorption also exists when *x* is equal to 0.^[^ ^21^ ^]^ While absorption covering from 430–600 nm of Nb_2_O_5_:*x*Pr^3+^ (*x* = 0.02, 0.05, 0.08) phosphors originates from the ^3^H_4_→^3^P_2_/^3^H_4_→^3^P_1_/^3^H_4_→^3^P_0_/^3^H_4_→^1^D_2_ transitions of Pr^3+^ ions, respectively.^[^ ^22^ ^]^ It is worth noting that the transition location of Pr^3+^ shows no change as the doped content increases due to the characteristics of *f*‐*f* transition.^[^ ^23^ ^]^ These results are also in accordance with the excitation spectra in Figure S4 (Supporting Information). Besides, the optical band gap of Nb_2_O_5_:*x*Pr^3+^ (*x* = 0, 0.02, 0.05, 0.08) samples could be calculated by the following Kubelka‐Munk equations:^[^ ^15^, ^24^ ^]^

(1) $${\left[F\left(R\infty \right)hv\right]}^{1/2}=A\left(hv-E_{g}\right)$$

(2) $$F\left(R\infty \right)={\left(1-R\right)}^{2}/2R$$

where *F(R)* is absorption, *R* is reflectance coefficient, *D* is absorption constant, *hv* is photon energy, and *E_g_* is optical band gap. The calculated *E_g_* of Nb_2_O_5_:*x*Pr^3+^ (*x* = 0, 0.02, 0.05, 0.08) samples are all ≈3.13 eV (Figure 3b), suggesting that the small amount of Pr^3+^ ions do not affect the bandgap. Besides, the calculated value of bandgap (Figure 3c) via DFT is 3.01 eV, slightly smaller than the experimental value, which is ascribed to aninherent deficiency of the computation method.^[^ ^25^ ^]^

**Figure 4:**
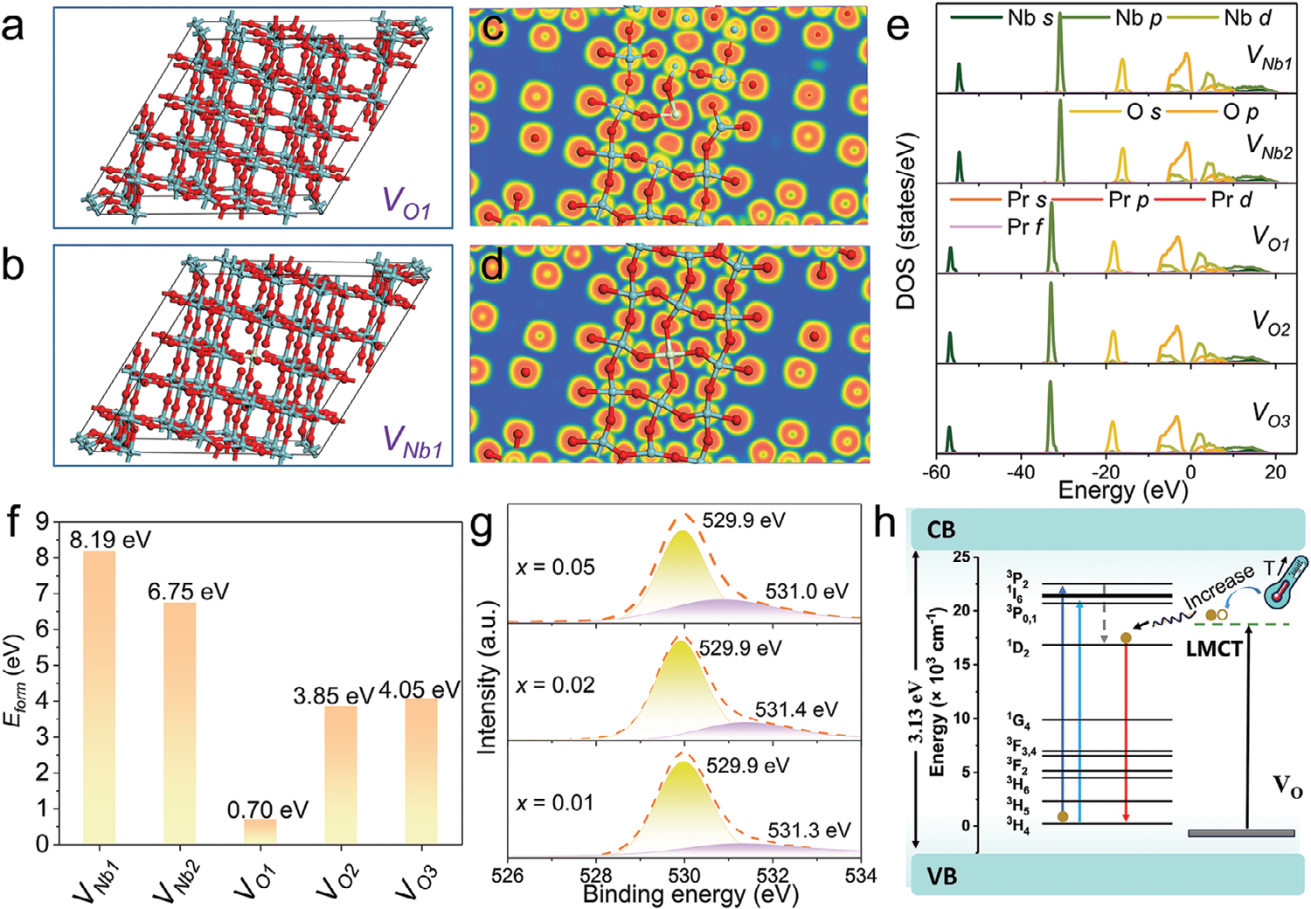
a) Crystal structure of Nb_2_O_5_:Pr^3+^ with (a) *V_O1_* and b) *V_Nb1_* in the lattice via DFT calculation. 3D ELF maps of Nb_2_O_5_:Pr^3+^ with c) *V_O1_* and d) *V_Nb1_*. e) Projected electronic densities of states and f) *E_form_* of five types of vacancies in the lattice. g) XPS spectra of O 1*s* and fitting results for Nb_2_O_5_:Pr^3+^ phosphors. h) Schematic illustration for the mechanism of ultra‐narrow band red emission and the anti‐thermal quenching at 80–280 K.

Photoluminescence excitation (PLE) and photoluminescence (PL) spectra of Nb_2_O_5_:*x*Pr^3+^ (0 ≤ *x* ≤ 0.08) samples are shown in Figure S4 (Supporting Information), it can be observed that the host (*x* = 0) does not emit light and the maximal emission intensity is achieved at *x* = 0.01. PLE spectra exhibit a major band (300–500 nm) centered at ≈398 nm when monitored at *λ*_em_ = 612 nm, and the strength of the major band is much greater than the ^3^H_4_→^3^P_2_/^3^H_4_→^3^P_1_/^3^H_4_→^3^P_0_ transition. Under the excitation of 398 nm, PL spectra present a single ultra‐narrow‐band emission at 612 nm with FWHM of only 19 nm, the red emission is attributed to the ^1^D_2_→^3^H_4_ transition of Pr^3+^ ions, and no emission peaks from ^3^P*_j_* (*j* = 0, 1, 2) to ^3^H_4_ transition are observed (Figure 3d). Table S2 (Supporting Information) lists the FWHM of Nb_2_O_5_:Pr^3+^ compared with other red commercial phosphors. The FWHM of classical red narrow‐band Sr[LiAl_3_N_4_]:Eu^2+^ and Sr[Li_2_Al_2_O_2_N_2_]:Eu^2+^ phosphors for LCD application are ≈50 nm, indicating the potential practical application of the narrower Nb_2_O_5_:Pr^3+^.^[^ ^1^, ^9^ ^]^ The symmetrical red emission with FWHM of 19 nm is even comparable to CsPbI_3_ quantum dot, and the former has excellent chemical stability and environmental friendliness.^[^ ^26^ ^]^ In Figure S4c (Supporting Information), the peak position and FWHM hardly change with the doped content. For Pr^3+^ doped phosphors, multiple linear peaks in green and red regions are common on account of the typical *f*‐*f* transition. However, the single ultra‐narrow‐band emission of Pr^3+^ is very different from the reported Pr^3+^ activated phosphors.^[^ ^27^ ^]^ It is speculated that the single narrow‐band emission is mainly related to the vacancies in the matrix like the reported CaTiO_3_:Pr^3+^ system.^[^ ^28^ ^]^ The highly pure red light is crucial for high‐quality warm white LED and display applications. In Figure 3e, the color purity is calculated via the following equation:^[^ ^11^, ^29^ ^]^

(3) $$\mathrm{color}\;\mathrm{purity}=\frac{\sqrt{{\left(x-x_{i}\right)}^{2}+{\left(y-y_{i}\right)}^{2}}}{\sqrt{{\left(x_{d}-x_{i}\right)}^{2}+{\left(y_{d}-y_{i}\right)}^{2}}}$$

where (*x*, *y*) is the CIE color coordinates of the phosphors, (*x_i_*, *y_i_*) is the white light source with CIE color coordinate (0.3333, 0.3333), and (*x_d_*, *y_d_*) is the color coordinate corresponding to the monochromatic light source. The color purities of Nb_2_O_5_:*x*Pr^3+^ (0.005 ≤ *x* ≤ 0.08) samples are ≈98%±1%, indicating the enormous potential in display application. Figure S5 (Supporting Information) shows the CIE coordinates of Nb_2_O_5_:*x*Pr^3+^ (0.005 ≤ *x* ≤ 0.08) samples. It can be seen that the CIE coordinates are basically at the same position (0.67, 0.33) and have not been changed with variation of Pr^3+^ concentration. Besides, the photoluminescence decay curves of Nb_2_O_5_:*x*Pr^3+^ (0.005 ≤ *x* ≤ 0.08) phosphors monitored at *λ*_em_ = 612 nm are collected in Figure S5 (Supporting Information), the calculated lifetime values are 3.7174, 5.5730, 4.6717, 3.9087, 3.3535, 3.2596, and 2.4345 ms, respectively. The millisecond‐level lifetime is ascribed to the forbidden *f*‐*f* transition of Pr^3+^ ions.^[^ ^30^ ^]^ With the increase of doped content, the luminescent lifetime first increases, reaching its maximum value at *x* = 0.01, and then begins to reduce. It is known that energy transfer from vacancies to Pr^3+^ and interaction between Pr^3+^ ions have the opposite effect on the lifetime. The doped content is relatively low, the interaction between Pr^3+^ ions is strong, and the large concentration quenching makes the PL lifetime become shorter. In Figure 3f, temperature‐dependent PLE and PL spectra are measured to investigate the photophysical properties of Nb_2_O_5_:0.01Pr^3+^ phosphor. As increasing temperature from 80 to 280 K, the intensity of the absorption band cover from 240 to 400 nm presents a linear increasing trend, while the intensity of ^3^H_4_ to ^3^P*_j_* (*j* = 0, 1, 2) transitions decreases due to thermal quenching effect. It means that the absorption of host is more efficient at higher temperatures because of the thermally activated exciton hopping migration.^[^ ^21^ ^]^ Under the excitation at 398 nm, PL spectra show an anti‐thermal quenching effect, the intensity presents an unexpected 12.5‐fold enhancement from 80 to 280 K. We think energy transfer from vacancies to luminescent center accounts for the enhancement. PL decay curves and fitting results of Nb_2_O_5_:0.01Pr^3+^ at 80 K are shown in **Figure** **4**g, the lifetime is evaluated to be 0.08 ms, which is smaller than the value at 298 K and in consistent with the emission intensity results. We also tested the PL decay curves of Nb_2_O_5_:0.03Pr^3+^ sample as a function of temperature in Figure S7 (Supporting Information), the energy that transfers from vacancies to Pr^3+^ increases as the temperature increases from 80 to 280 K, thus the PL lifetime continuously increases. These results confirm the existence of the energy transfer from vacancies to the luminescent center.

DFT calculation is conducted to further study vacancy types and electron properties of Nb_2_O_5_:*x*Pr^3+^ system. The calculation is performed with the Perdew‐Burke‐Ernzerhof (PBE) functional based on generalized gradient approximation (GGA).^[^ ^31^ ^]^ The double numerical atomic orbital augmented by a polarization function is chosen as the basis set. The crystal structure and projected electronic densities of states of pristine Nb_2_O_5_:Pr^3+^ without vacancies in the lattice via DFT calculation are shown in Figure S8 (Supporting Information). The bottom of the conduction band (CB) is mainly contributed by O *p* and Nb *d* states and the top of the valence band (VB) is dominated by O *s* and Nb *p* states. The location of the CB minimum and VB maximum at different points indicates an indirect bandgap. Due to the low content of the doped Pr^3+^ ions, it has a limited contribution to band formation. As mentioned above, Pr^3+^ ions mainly occupy Nb sites, to maintain charge balance, the heterovalent substitution can cause niobium vacancies. Besides, oxygen vacancies are also easily observed in high‐temperature solid‐state reactions under an air atmosphere.^[^ ^32^ ^]^ Figure 4a,b and Figure S9 (Supporting Information) present the crystal structure of Nb_2_O_5_:Pr^3+^ with oxygen vacancies and niobium vacancies in the lattice, we mainly considered three oxygen vacancies (*V_O1_, V_O2_, V_O3_*) and two niobium vacancies (*V_Nb1_*, *V_Nb2_*) which are most likely to formed.^[^ ^33^ ^]^ The obtained structural information via DFT calculation is consistent with the Rietveld refinement results. In order to study the influence of vacancies on the Pr^3+^ luminescence behavior in Nb_2_O_5_:*x*Pr^3+^ system, the ELF plots are analyzed with different vacancies in Figure 4c,d and Figure S9 (Supporting Information). When Pr^3+^ ions substitute Nb ions, many electrons will localize around the Pr atoms and fewer electrons will be concentrated on coordinated O atoms, the electron localization area around the Pr atom enlarges as the adjacent vacancy appears. Besides, by comparing the ELF maps with the five traps, it could be found that the electron localization area around the Pr atom enlarges and presents the biggest distortion as *V_O1_* appears. Thus, the unique ultra‐narrow‐band emission of Nb_2_O_5_:*x*Pr^3+^ is mainly related to the presence of inherent oxygen vacancies in the Nb_2_O_5_:*x*Pr^3+^ system. In Figure 4e and Figure S8 (Supporting Information), we calculated the electron structures of Nb_2_O_5_:Pr^3+^ without vacancies and with oxygen/niobium vacancies. According to the DFT results, the composition of the VB and CB has not changed significantly. However, Compared to niobium vacancies, the whole electron distribution of Nb_2_O_5_:Pr^3+^ with oxygen vacancies in the lattice shifts in the low‐energy direction by ≈2.0 eV. Moreover, electron transition energy levels of the Pr *s*, Pr *p*, Pr *d*, and Pr *f* orbits at CB and VB of Nb_2_O_5_:Pr^3+^ matrix are shown in Figure S10 (Supporting Information). The different energy level splitting in Pr *s* orbital is that the distances between *V_O2_* and the lattice site occupied by Pr^3+^ ion is the farthest compared to *V_O1_* and *V_O3_*. Therefore, the influence on the electronic structure of Pr is small, resulting in no energy level splitting in *V_O2_*. More importantly, the corresponding formation energies (*E_form_*) of the five types of vacancies are calculated theoretically to uncover which type most affects Pr^3+^ luminescence. By comparing the formation energy values in Figure 4f, it could be observed that the *E_form_* of oxygen vacancies is lower than the niobium vacancies. Obviously, the vacancy of *V_O1_* has the lowest *E_form_* of 0.70 eV after the substitution of Pr^3+^ ions for Nb sites. The results confirm that the form of oxygen vacancies is preferable and the results agree well with the ELF results.

XPS measurement is used to confirm the existence of oxygen vacancies in the experiment. Figure 4g presents the HR‐XPS spectra of O 1*s* and fitting results of Nb_2_O_5_:*x*Pr^3+^ phosphors, the O 1*s* orbit could be fitted by two Gaussian peaks that are centered at ≈529.9 and ≈530.3 eV. In theory, the XPS signals would be observed at one bind energy because of the only Nb‐O bonds in the lattice. Electron energy corresponding to the oxygen vacancies in the lattice is ≈531.1 eV according to the previous reports, which is close to our measured experimental values.^[^ ^34^ ^]^ The second Gaussian peak (≈531.3 eV) is ascribed to the interstitial oxygen vacancies. These results confirm that oxygen vacancies indeed exist in the phosphors. The photophysical process for single ultra‐narrow‐band red emission of Nb_2_O_5_:Pr^3+^ is shown in Figure 4h, electrons jump to excited states after absorbing energy. According to DR spectra, the main absorption (240–400 nm) is related to the electron transfer from O^2–^ to Nb^5+^, and temperature‐dependent PLE spectra show that the absorption band (240‐400 nm) presents a linear increasing trend from 80 to 280 K. Thus, we need to consider the intermediate ligand‐to‐metal charge transfer (LMCT) state here, like the reported CaTiO_3_:Pr^3+^ phosphor.^[^ ^35^ ^]^Based on DFT and HR‐XPS results, oxygen vacancies indeed exist in the matrix lattice. These oxygen vacancies can regulate the electron hole recombination energy, causing the LMCT state to be located below ^3^P_0_ and slightly higher than ^1^D_2_, The energy could transfer directly to the ^1^D_2_ level of Pr^3+^, while the transition of the ^3^P*_j_* to GS is completely quenched. It means the direct host‐to‐dopant energy transfer selectively populates the ^1^D_2_ red luminescent state of Pr^3+^ and bypasses the ^3^P_0_ greenish‐blue emitter, resulting in the single ^1^D_2_→^3^H_4_ transition and emitting a single ultra‐narrow‐band red emission. Besides, When the temperature rises from 80 to 200 K, the energy from oxygen vacancies to ^1^D_2_ level increases, which explains the reason for 12.5‐fold emission enhancement.

**Figure 5:**
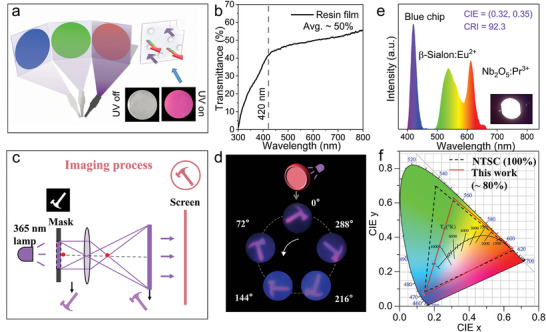
a) Demonstration of TD screen design based on Nb_2_O_5_:Pr^3+^ phosphor. The inset photographs show transparent resin film based on Nb_2_O_5_:Pr^3+^ phosphor under natural light and 365 UV lamp. b) Transmittance curve of as‐prepared resin film. c) Schematic diagram of display demonstration for an example of “hammer”. d) Luminescent images of “hammer” on the screen under a dark environment. e) Electroluminescence spectra of white LED device (420 nm GaN chip + *β*‐SiAlON:Eu^2+^ + Nb_2_O_5_:Pr^3+^). f) CIE chromaticity coordinates of NTSC standard (dark dotted line) and obtained results (red line).

**Figure** **5**a presents a demonstration of TD screen design based on narrow‐band red Nb_2_O_5_:Pr^3+^ phosphor. The inset photographs show that Nb_2_O_5_:Pr^3+^ phosphor embedded in layered resin film is transparent under natural light and exhibits bright red light under 365 UV lamps. The as‐prepared screen film has a size of 3.0 × 3.0 × 0.15 cm^−3^, and can be customized according to the size of the model. Figure S11 (Supporting Information) presents the fabrication procedure of the screen film, the method is similar to the previously reported work and the specific details can be found in the experimental section.^[^ ^36^ ^]^ After 2 hours of grinding and sonication treatment, the phosphor is spread between two layers of resin. The entire layer of phosphor can be filled between two layers of resin, the transparency is related to the particle size and the ratio of resin and phosphor. As the amount of feeding phosphor increases, the luminescent intensity of the film increases, but the transparency decreases. Based on the comprehensive luminescence intensity and transparency performance, the optimal molar ratio of resin and phosphor is 50:1. In Figure 5b, the transmittance curve indicates that as‐prepared resin film (50:1) presents good transparency with an average visible‐light (420–800 nm) transmittance (*T* ≈ 50%). The obtained *T* value is smaller than the reported NaLi_3_SiO_4_:Eu^2+^ TD screen film (60%), but better than an electroluminescent screen with transparent electronics (< 50%).^[^ ^36^, ^37^ ^]^ The Nb_2_O_5_:Pr^3+^ particles may need high‐power mechanical grinding and film thickness reduction to further improve transparency. To simulate the application of the transparent film produced in the display field, a simple TD prototype (Figure 5c) is built by assembling components such as a light source, image regulator, “hammer” shaped mask, convex lens, and transparent film. Then it is projected on screen film in an enlarged and inversed form through a convex lens. At last, the “hammer” shaped excitation light selectively light up the Nb_2_O_5_:Pr^3+^ phosphor in the transparent film, achieving the imaging of the red “hammer” shaped pattern on the screen. The image regulator, constructed by a rotatable wheel and carved masks riveted on the circumference, is used for easy image adjustment. The “hammer” shaped pattern with several angles on the screen film at dark environment is shown in Figure 5d, which vividly depicts that the red‐emitting “hammer” appears in sequence. In Figure 5e, the white LED is fabricated by coating the 420 nm GaN chip with the mixture of commercial narrow‐band *β*‐SiAlON:Eu^2+^ (green) and as‐prepared Nb_2_O_5_:Pr^3+^ phosphor. The warm white LED with low CCT = 5552 K, high CRI = 92.3, and CIE color coordinate of (0.32, 0.35) could be gained. However, due to the relatively low photoluminescence quantum yield of Nb_2_O_5_:Pr^3+^ phosphor, the luminous efficacy of LED devices needs to be further increased. The device could cover the color space of the 80% NTSC in CIE 1931. According to the coordinate points in Figure 5f, it can be seen that the area has not reached 95% is mainly due to the coordinate point of commercial *β*‐SiAlON:Eu^2+^ does not meet the standard value.^[^ ^7^ ^]^ These results indicate the Nb_2_O_5_:Pr^3+^ phosphor has the potential application in the display field.

## 3 Conclusion

In summary, we synthesized a series of Nb_2_O_5_:*x*Pr^3+^ (0.005 ≤ *x* ≤ 0.08) phosphors with nanorod‐like structure. PL spectra present a single ultra‐narrow‐band emission at 612 nm with FWHM of only 19 nm under n‐UV excitation. The wide excitation area can match well with n‐UV LED chips, and the FWHM is smaller than classically commercial red phosphors, such as Sr[LiAl_3_N_4_]:Eu^2+^ and Sr[Li_2_Al_2_O_2_N_2_]:Eu^2+^. Besides, the intensity of PL spectra presents an unexpected 12.5‐fold enhancement from 80 to 280 K, which is attributed to energy transfer from vacancies to the luminescent center. Absorption behavior, PL decay curves, DFT calculation are conducted to analyze electron properties and the luminescent mechanism of Nb_2_O_5_:*x*Pr^3+^ system. It could be found that the *E_form_* of oxygen vacancies is lower than the niobium vacancies and *V_O1_* has the lowest *E_form_* of 0.70 eV. Besides, ELF results indicate that the electron localization area around the Pr atom enlarges and presents the biggest distortion as *V_O1_* appears. In the experiment, XPS measurement confirms the existence of oxygen vacancies. The reason for the single narrow red emission is because that oxygen vacancies can influence the electron hole recombination energy, causing the LMCT state to be located below ^3^P_0_ and slightly higher than ^1^D_2_. The energy could transfer directly to the ^1^D_2_ level of Pr^3+^, while the transition of the ^3^P*_j_* to GS is completely quenched. Finally, the fabrication and evaluation of TD screen (transparency ≈50%, color purity ≈98%) and LED device (large color gamut, 80% NTSC) indicates the potential application in the transparent display field.

## Conflict of Interest

The authors declare no conflict of interest.

## Supporting information



Supporting Information

## Data Availability

The data that support the findings of this study are available from the corresponding author upon reasonable request.
