# Supplementary material for: Designing Ultra‐Narrow‐Band Red Phosphor via Oxygen Vacancy Engineering for Transparent Display Application
Source: Adv Sci (Weinh). 2025 Feb 17;12(14):2416761. doi: 10.1002/advs.202416761 (PMC11984928; doi:10.1002/advs.202416761)
Supplement: Supplementary file 1 — Supporting Information [file ADVS-12-2416761-s001.docx]

Copyright WILEY-VCH Verlag GmbH & Co. KGaA, 69469 Weinheim, Germany, 2024.

Supporting Information

***Designing ultra-narrow-band red phosphor via oxygen vacancy engineering for transparent display application***

*Wei Wang*,* *Yi Wei, Hang Yang, Jinxuan Sun, Fuyan Su, Hanrui Liao,* *Hua Zou*, Mingrui Li*, and* *Guogang Li**

Dr. W. Wang, F. Su, Prof. H. Zou

College of Physics and Optoelectronic Engineering, Hainan University, 58 Renmin Avenue, Haikou 570228, China

E-mail: wangweisw@hainanu.edu.cn; zouhua@hainanu.edu.cn

Dr. Y. Wei, H. Yang, J. Sun, H. Liao, Prof. G. Li

Faculty of Materials Science and Chemistry, China University of Geosciences, 388 Lumo Road, Wuhan 430074, P. R. China
E-mail: ggli@cug.edu.cn

Dr. M. Li

Department of Chemistry, Southern University of Science and Technology, Shenzhen 518055, Guangdong, P. R. China

E-mail: lmr@bjmu.edu.cn

**Experimental Section**

***Materials and Preparation*:** A series of Nb_2_O_5_:*x*Pr^3+^ (0 ≤ *x* ≤ 0.08) samples are prepared by a traditional high-temperature solid-state method. Niobium pentaoxide (Nb_2_O_5_, 99.99%) and praseodymium oxide (Pr_6_O_11_, 99.99%) were purchased from Aladdin and used without further purification. The raw materials were weighed according to the stoichiometric amounts of the required cationic sources and ground thoroughly in an agate mortar for 50 minutes. Then the obtained samples were put into an alumina crucible and sintered at 1300 ˚C for 10 h under air atmosphere.

***Fabrication of screen film and LED device*:** Firstly, the weighed epoxy resin and curing agent were mixed and thoroughly stirred, then the obtained mixture were poured into the customized circular mold. After drying in an oven at 60 °C for 6 hours, the mixture was solidified and could form a resin base. Then, epoxy resin, curing agent and Nb_2_O_5_:0.01Pr^3+^phosphor were weighed, the optimal molar ratio of epoxy resin and the Nb_2_O_5_:0.01Pr^3+^phosphor is 50:1. The mixture was thoroughly stirred and poured on resin base, then drying in an oven at 60 °C until it was solidified. The warm white LED device was fabricated by 420 nm GaN chip with the mixture of β-SiAlON:Eu^2+^ and Nb_2_O_5_:0.01Pr^3+^ phosphors. The process is adding phosphors into the epoxy resins and mixing thoroughly for 30 minutes. Then the obtained mixture was coated on the surface of 420 nm GaN chip and dried at 120 °C in an oven. The electroluminescence measurements were conducted under a voltage of 3 V and currents 20 mA on the HAAS 2000 photoelectric measuring system from EVERFINE.

***DFT calculation*:** All the calculated results were obtained by using the first-principle method through the DMol3 code. The All Electron Relativistic treatment was employed for relativistic effects which explicitly includes all electrons and applies some relativistic effects into the core. The calculation is performed using the density functional theory (DFT) with the Perdew-Burke-Ernzerhof (PBE) functional based on generalized gradient approximation(GGA). The double numerical atomic orbital augmented by a polarization function was chosen as the basis set. In the geometry structural optimization, T e convergence tolerances of energy, maximum force and displacement were set at ‘fine’ quality which are 1 × 10^−5^ Ha, 2 × 10^−3^ Ha Å−1, and 5 × 10^−3^ Å, respectively.

***Characterizations*.** Powder X-ray diffraction (PXRD) measurements are conducted on a D8 Focus diffractometer equipped with the Cu Kα (1.54 Å) radiation. The PXRD data for Rietveld refinements has a scanning speed of 1° per min in the 2θ range of 5°-90°. Rietveld refinements are performed with the General Structure Analysis System (GSAS) software. High-resolution transmission electron microscopy (HR-TEM) are performed on a FEI Tecnai G2 S-Twin equipped with a field emission gun operating at 200 kV. The morphology and elemental mapping are detected on a field emission scanning electron microscope (FE-SEM, S-4800, Hitachi). X-ray photoelectron spectroscopy (XPS) measurements are conducted with a Thermo Fischer ESCALAB 250 Xi, Raman spectra are obtained on Raman spectrometer (JYT6400) with a 1064 nm laser. The absorption spectra and transmittance spectra are detected on an UV-vis-NIR spectrophotometer (PE lambda 750). Temperature-dependent photoluminescence excitation (PLE) spectra, temperature-dependent photoluminescence (PL) spectra, and photoluminescence decay curves are monitored using a fluorescence spectrometer (Edinburgh FLS-1000).

**
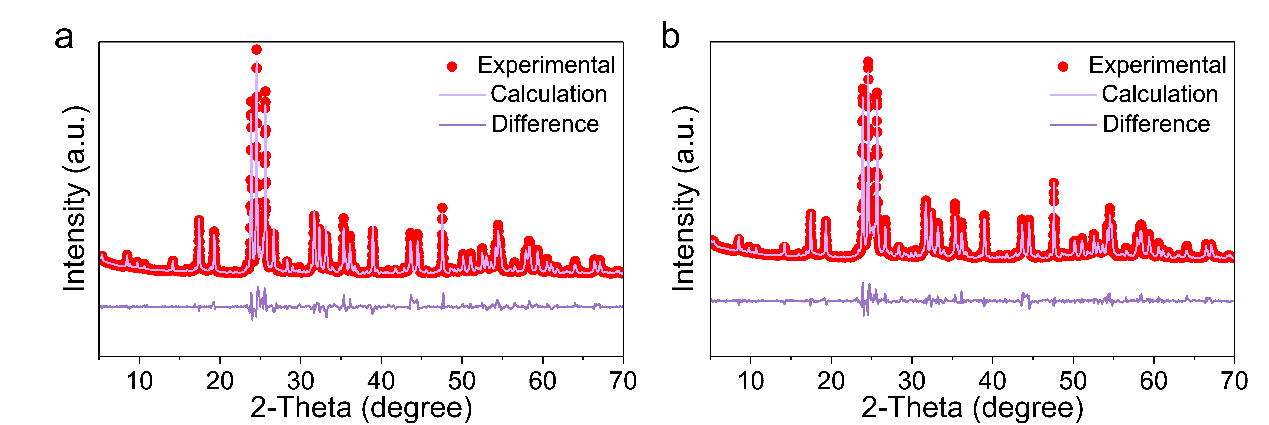
**

Figure S1 XRD Rietveld refinement of (a) Nb_2_O_5_ host and (b) Nb_2_O_5_:Pr^3+^ phosphor.

Table S1. Crystallographic parameters gained from Rietveld refinements for Nb_2_O_5_ host and Nb_2_O_5_:0.01Pr^3+^ phosphor.

|  | Nb_2_O_5_ host | Nb_2_O_5_:Pr^3+^ |
| --- | --- | --- |
| Crystal system | monoclinic | monoclinic |
| Space group | *P2* | *P2* |
| *a* [Å] | 21.1682(4) | 21.1722(8) |
| *b* [Å] | 3.8214(7) | 3.8255(3) |
| *c* [Å] | 19.3531(3) | 19.3621(6) |
| *V* [Å^3^] | 1358.15(4) | 1360.45(1) |
| *α* [°] | 90 | 90 |
| *γ* [°] | 119.8254(1) | 119.8289(1) |
| *β* [°] | 90 | 90 |
| *R*_wp_ [%] | 10.02% | 11.06% |
| *R*_p_ [%] | 7.57% | 7.98% |
| χ^2^ | 4.802 | 4.987 |


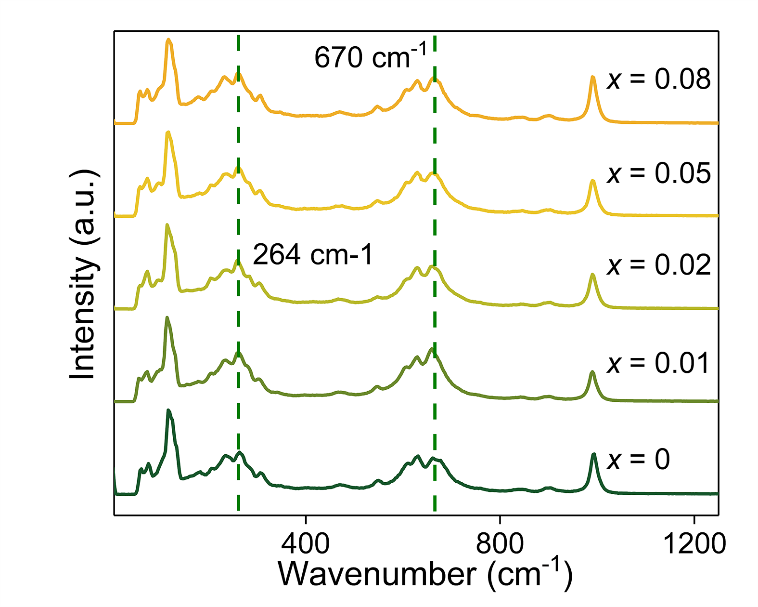


Figure S2 Raman spectra of Nb_2_O_5_:*x*Pr^3+^ (*x* =0, 0.01, 0.02, 0.05, 0.08) phosphors.


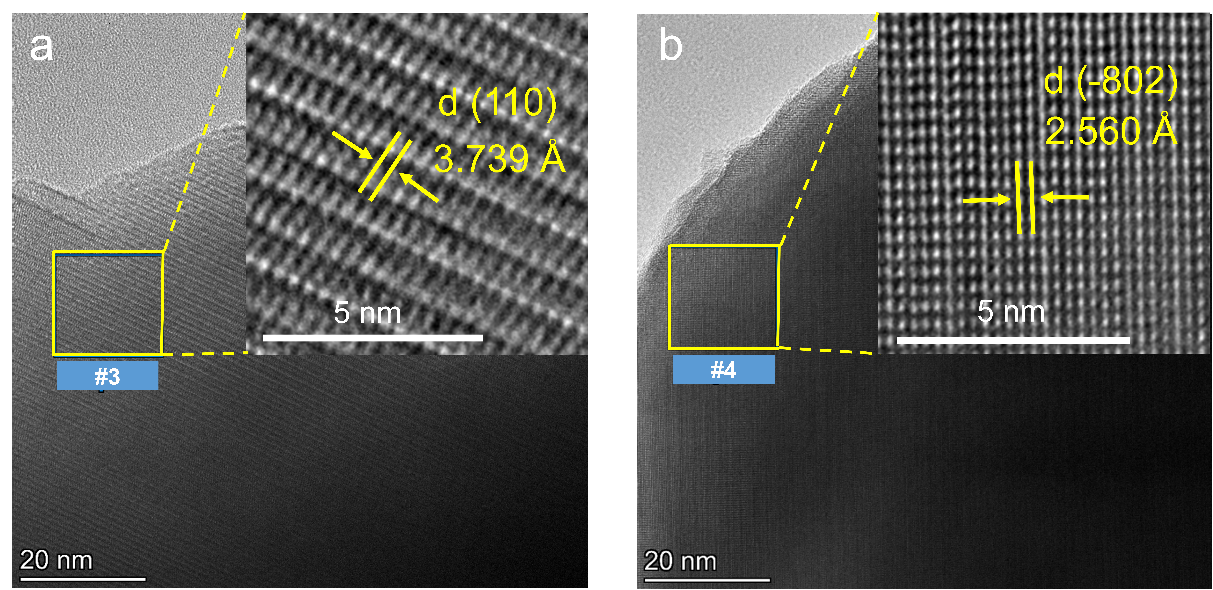


Figure S3 HR-TEM images and calculated lattice fringes of selected high-resolution area (a) #3 and (b) #4, respectively.


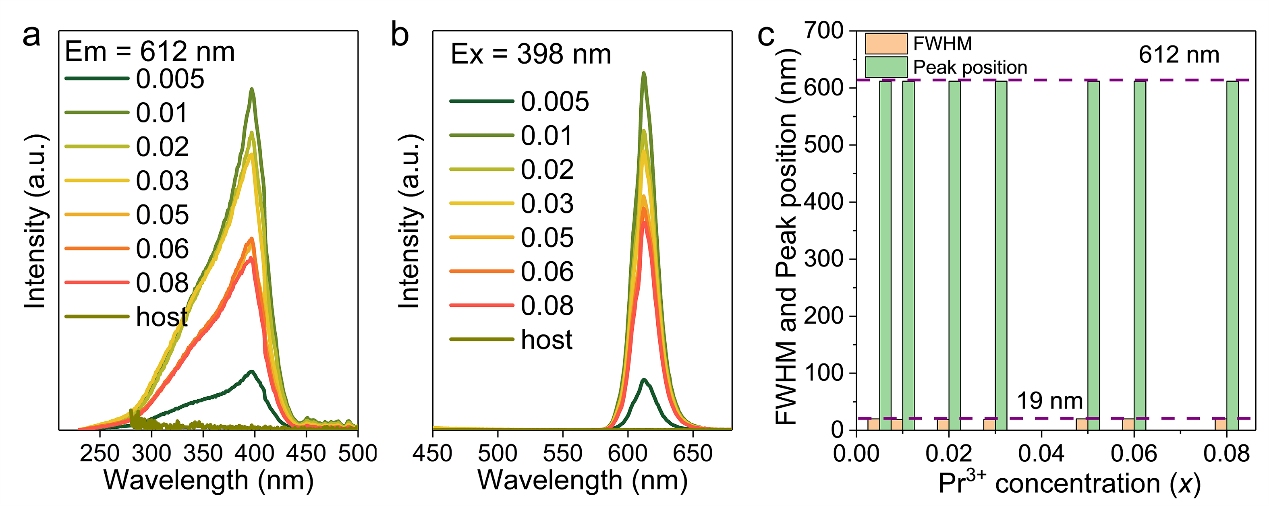


Figure S4 (a) PLE and (b) PL of Nb_2_O_5_:*x*Pr^3+^ (0 ≤ *x* ≤ 0.08) phosphors. (c) The FWHM and peak position of Nb_2_O_5_:*x*Pr^3+^ phosphors at various Pr^3+^ contents.

Table S2 Comparison of FWHM of classical red-emitting phosphors

| Samples | *E_m_* (nm) | FWHM (nm) | Ref |
| --- | --- | --- | --- |
| Sr[LiAl_3_N_4_]:Eu^2+^ | 650 | 50 | ^[1]^ |
| Sr[Li_2_Al_2_O_2_N_2_]:Eu^2+^ | 614 | 48 | ^[2]^ |
| Sr[Mg_3_SiN_4_]:Eu^2+^ | 615 | 43 | ^[3]^ |
| Ca[LiAl_3_N_4_]:Eu^2+^ | 668 | 60 | ^[4]^ |
| La_3−_*_x_*Ca_1.5_*_x_*Si_6_N_11_:Eu^2+^ | 587 | 60 | ^[5]^ |
| CaAlSiN_3_:Eu^2+^ | 630 | 86 | ^[6]^ |
| Ba[Mg_3_SiN_4_]:Eu^2+^ | 670 | 88 | ^[7]^ |
| Sr_4_[LiAl_11_N_14_]:Eu^2+^ | 670 | 85 | ^[8]^ |
| Sr_5_Si_7_P_2_N_16_:Eu^2+^ | 592 | 118 | ^[9]^ |
| Rb_3_YSi_2_O_7_:Eu^2+^ | 622 | 124 | ^[10]^ |
| Sr_2_Sc_0.5_Ga_1.5_O_5_:Eu^2+^ | 614 | 86 | ^[11]^ |
| Sr_3_TaO_5.5_:Eu^2+^ | 620 | ~75 | ^[12]^ |
| BaSrGa_4_O_8_:Eu^2+^ | 670 | 140 | ^[13]^ |
| K_3_YSi_2_O_7_: Eu^2+^ | 622 | ~104 | ^[14]^ |
| Cs_3_GdGe_3_O_9_:Eu^3+^ | 611 | Multiple line emissions | ^[15]^ |
| Y_2_O_3_:Eu^3+^ | 612 | Multiple line emissions | ^[16]^ |
| K_2_SiF_6_:Mn^4+^ | 631 | Multiple line emissions | ^[17]^ |
| K_2_TiF_6_:Mn^4+^ | 631 | Multiple line emissions | ^[18]^ |
| CsPbI_3_ | 630 | 5-20 | ^[19]^ |


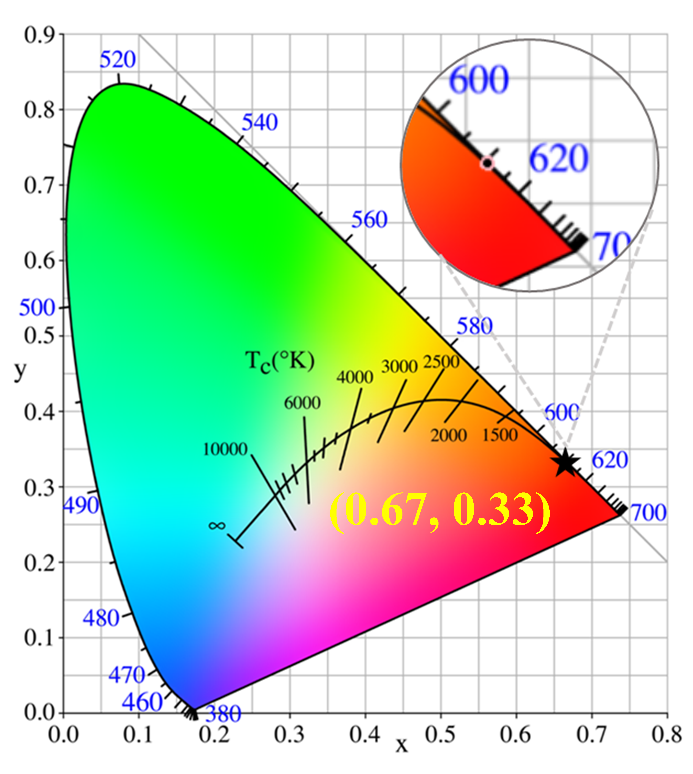


Figure S5 The CIE chromaticity coordinates of Nb_2_O_5_:Pr^3+^ phosphors.


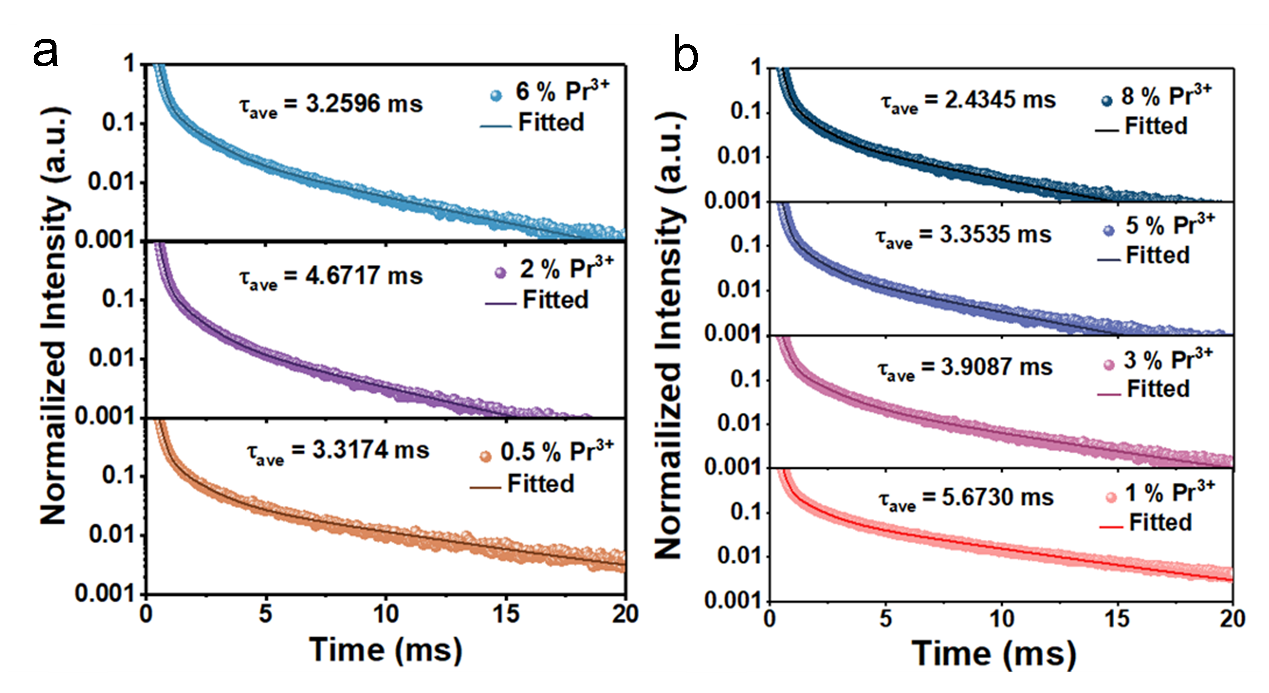
 Figure S6 PL decay curves and fitting results of Nb_2_O_5_:*x*Pr^3+^ (0.005 ≤ *x* ≤ 0.08) phosphors at room temperature.


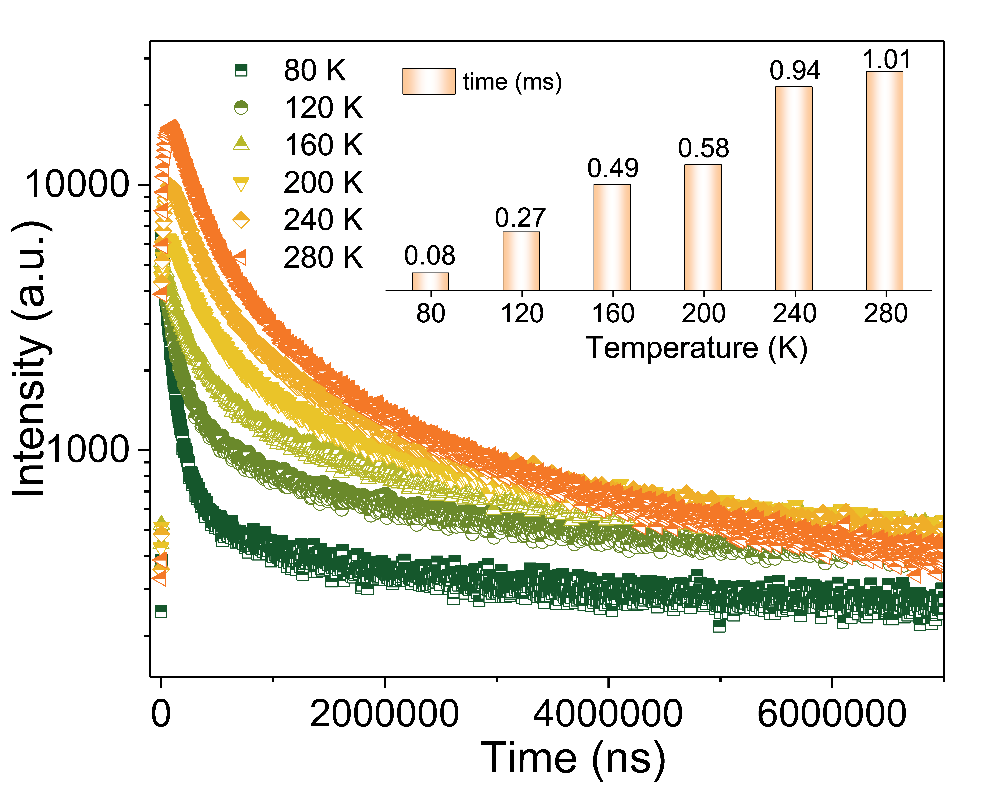


Figure S7 The photoluminescence decay lifetime of Nb_2_O_5_:0.03Pr^3+^ phosphor as a function of temperature (80-280 K)


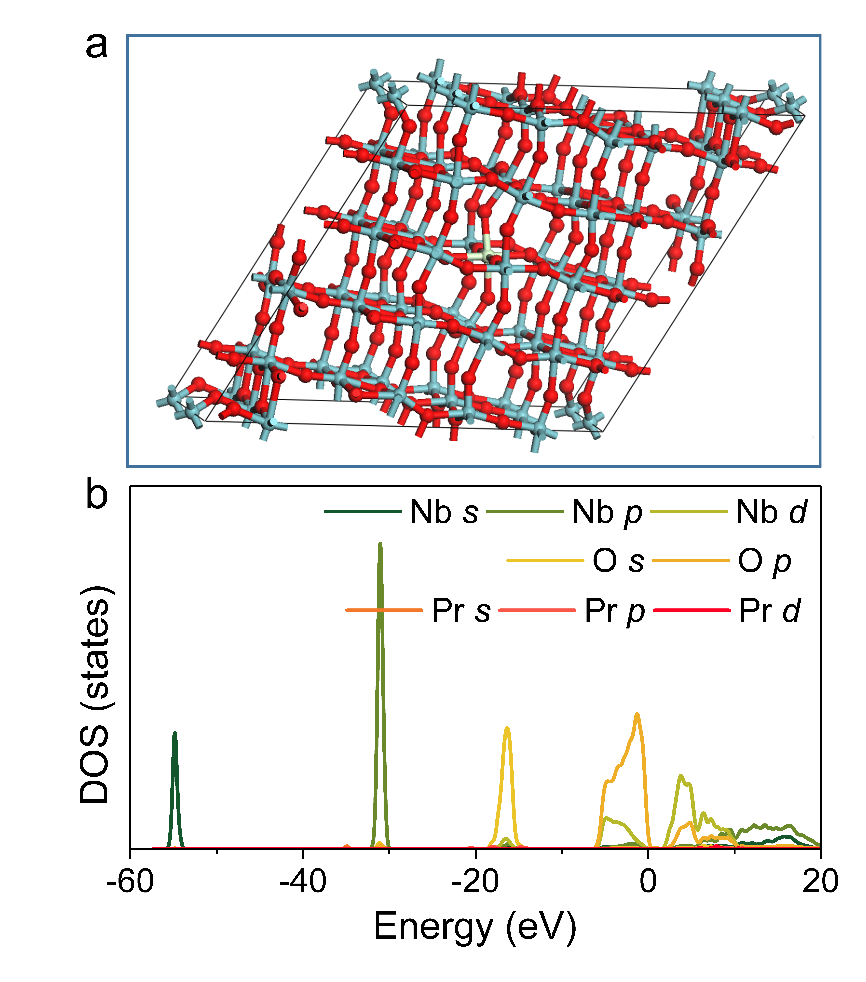


Figure S8 (a) Crystal structure and (b) projected electronic densities of states of Nb_2_O_5_:Pr^3+^ without vacancies in the lattice via DFT calculation.


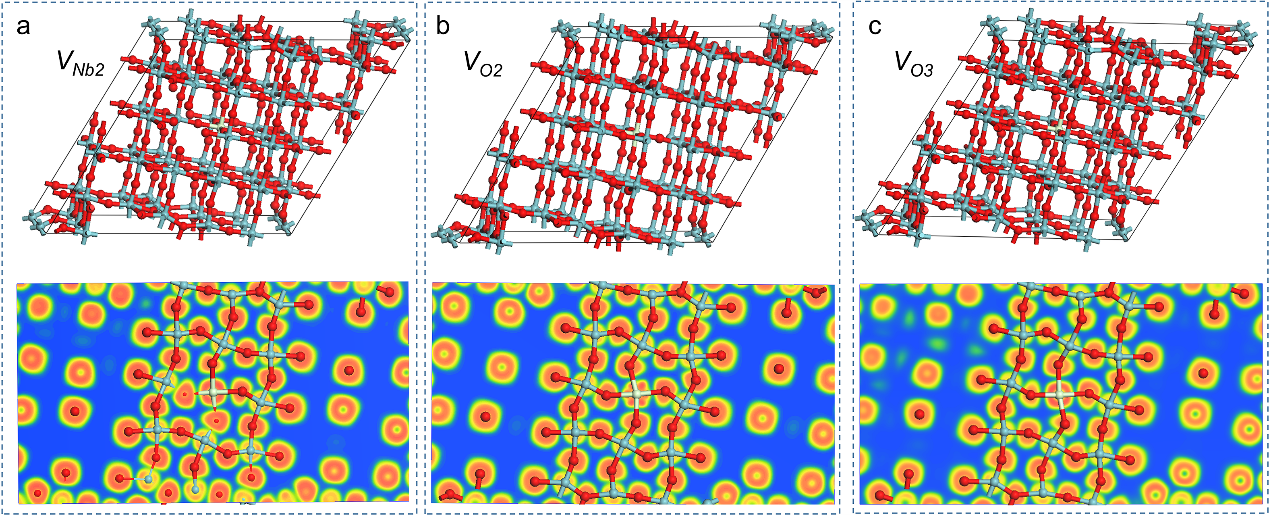


Figure S9 Crystal structure (top) and 3D ELF maps (bottom) of Nb_2_O_5_:Pr^3+^ with (a) *V_Nb2_*, (b) *V_O2_,* and (c) *V_O3_* in the lattice via DFT calculation.


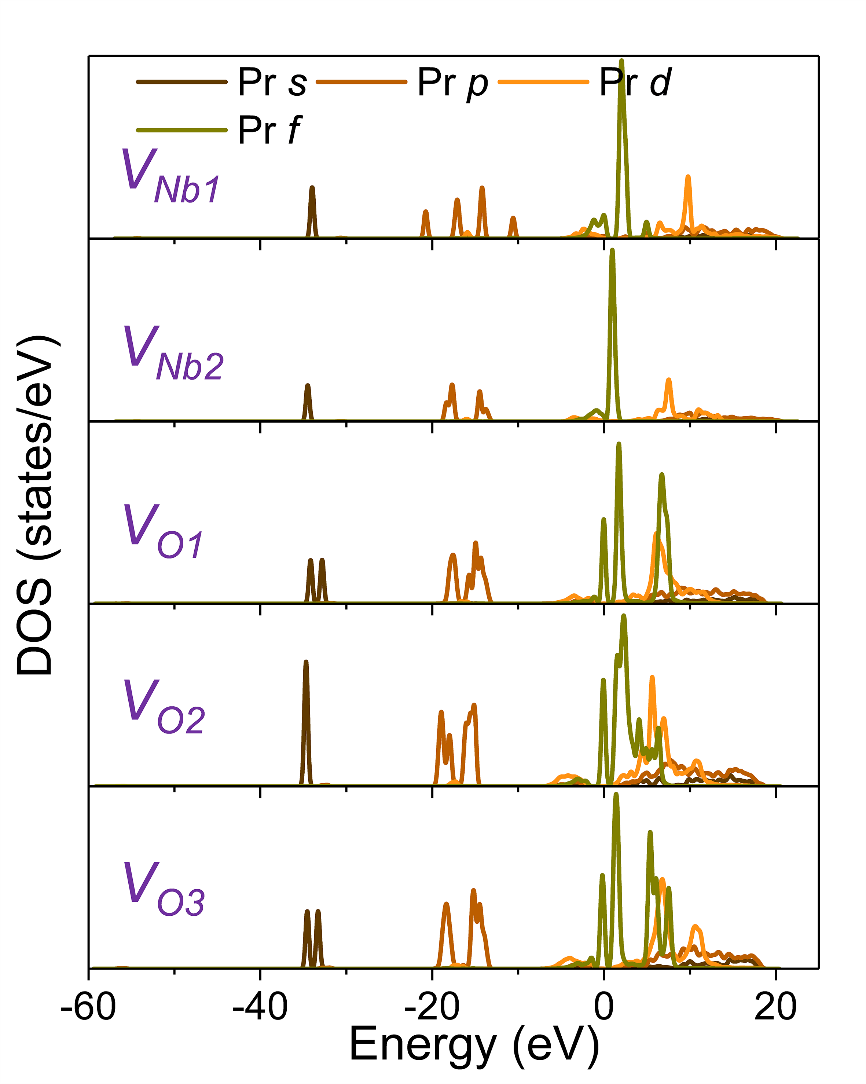


Figure S10 Projected electronic densities of states of different vacancies in the lattice.


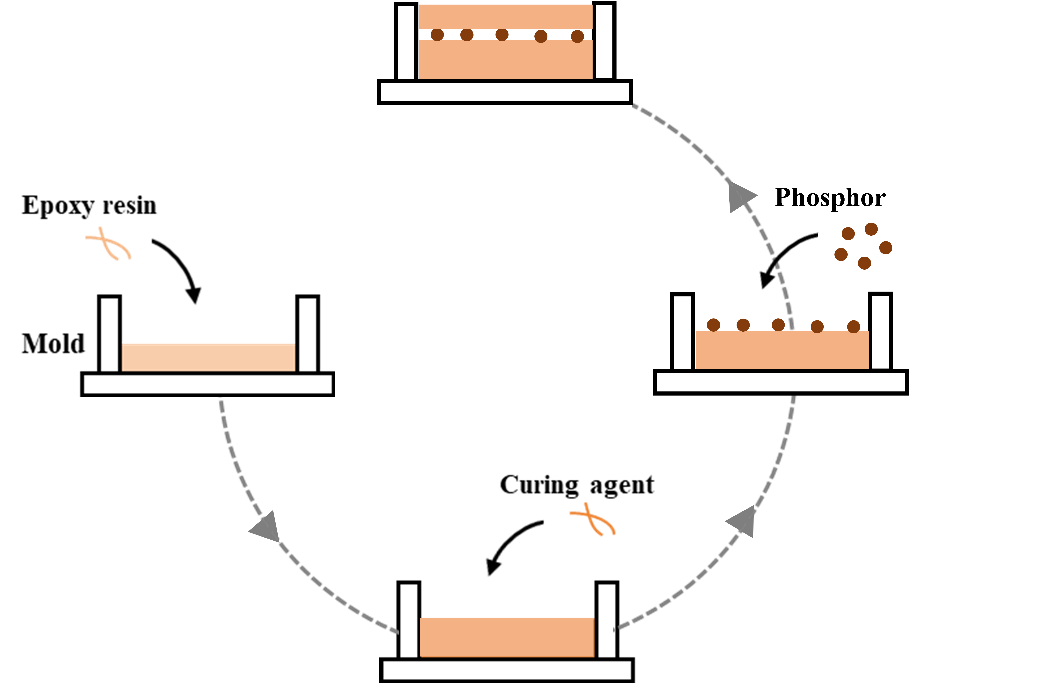


Figure S11 The process for layered screen film fabrication based on Nb_2_O_5_:Pr^3+^ phosphor.

**References**

[1] P. Pust, V. Weiler, C. Hecht, A. Tücks, A. S. Wochnik, A.-K. Henß, D. Wiechert, C. Scheu, P. J. Schmidt, W. Schnick, *Nat. Mater.* **2014**, *13*, 891-896.

[2] G. J. Hoerder, M. Seibald, D. Baumann, T. Schröder, S. Peschke, P. C. Schmid, T. Tyborski, P. Pust, I. Stoll, M. Bergler, C. Patzig, S. Reißaus, M. Krause, L. Berthold, T. Höche, D. Johrendt, H. Huppertz, *Nat. Commun.* **2019**, *10*, 1824.

[3] S. Schmiechen, H. Schneider, P. Wagatha, C. Hecht, P. J. Schmidt, W. Schnick, *Chem. Mater.* **2014**, *26*, 2712-2719.

[4] P. Pust, A. S. Wochnik, E. Baumann, P. J. Schmidt, D. Wiechert, C. Scheu, W. Schnick, *Chem. Mater.* **2014**, *26*, 3544-3549.

[5] C. Maak, D. Durach, C. Martiny, P. J. Schmidt, W. Schnick, *Chem. Mater.* **2018**, *30*, 3552-3558.

[6] Y.-T. Tsai, C.-Y. Chiang, W. Zhou, J.-F. Lee, H.-S. Sheu, R.-S. Liu, *J. Am. Chem. Soc.* **2015**, *137*, 8936-8939.

[7] L. Wang, R.-J. Xie, T. Suehiro, T. Takeda, N. Hirosaki, *Chem. Rev.* **2018**, *118*, 1951-2009.

[8] D. Wilhelm, D. Baumann, M. Seibald, K. Wurst, G. Heymann, H. Huppertz, *Chem. Mater.* **2017**, *29*, 1204-1209.

[9] M. Dialer, M. M. Pointner, S. L. Wandelt, P. Strobel, P. J. Schmidt, L. Bayarjargal, B. Winkler, W. Schnick, *Adv. Optical Mater.* **2023**, 2302668.

[10] J. Qiao, L. Ning, M. S. Molokeev, Y. C. Chuang, Q. Zhang, K. R. Poeppelmeier, Z. Xia, *Angew. Chem. Inter. Ed.* **2019**, *58*, 11521-11526.

[11] Z. Yang, Y. Zhou, J. Qiao, M. S. Molokeev, Z. Xia, *Adv. Optical Mater.* **2021**, *9*, 2100131.

[12] Z. Yang, T. de Boer, P. M. Braun, B. Su, Q. Zhang, A. Moewes, Z. Xia, *Adv. Mater.s* **2023**, *35*, 2301837.

[13] Y. Zhu, X. Wang, J. Qiao, M. S. Molokeev, H. C. Swart, L. Ning, Z. Xia, *Chem. Mater.* **2023**, *35*, 1432-1439.

[14] J. Qiao, M. Amachraa, M. Molokeev, Y.-C. Chuang, S. P. Ong, Q. Zhang, Z. Xia, *Chem. Mater.* **2019**, *31*, 7770-7778.

[15] P. Dang, G. Li, X. Yun, Q. Zhang, D. Liu, H. Lian, M. Shang, J. Lin, *Light Sci. Appl.* **2021**, *10*, 29.

[16] J. Yang, Z. Quan, D. Kong, X. Liu, J. Lin, *Cryst. Growth Des.* **2007**, *7*, 730-735.

[17] L. Huang, Y. Zhu, X. Zhang, R. Zou, F. Pan, J. Wang, M. Wu, *Chem. Mater.* **2016**, *28*, 1495-1502.

[18] H. Zhu, C. C. Lin, W. Luo, S. Shu, Z. Liu, Y. Liu, J. Kong, E. Ma, Y. Cao, R.-S. Liu, X. Chen, *Nat. Commun.* **2014**, *5*, 4312.

[19] A. Dey, J. Ye, A. De, E. Debroye, S. K. Ha, E. Bladt, A. S. Kshirsagar, Z. Wang, J. Yin, Y. Wang, L. N. Quan, F. Yan, M. Gao, X. Li, J. Shamsi, T. Debnath, M. Cao, M. A. Scheel, S. Kumar, J. A. Steele, M. Gerhard, L. Chouhan, K. Xu, X. G. Wu, Y. Li, Y. Zhang, A. Dutta, C. Han, I. Vincon, A. L. Rogach, A. Nag, A. Samanta, B. A. Korgel, C. J. Shih, D. R. Gamelin, D. H. Son, H. Zeng, H. Zhong, H. Sun, H. V. Demir, I. G. Scheblykin, I. Mora-Sero, J. K. Stolarczyk, J. Z. Zhang, J. Feldmann, J. Hofkens, J. M. Luther, J. Perez-Prieto, L. Li, L. Manna, M. I. Bodnarchuk, M. V. Kovalenko, M. B. J. Roeffaers, N. Pradhan, O. F. Mohammed, O. M. Bakr, P. Yang, P. Muller-Buschbaum, P. V. Kamat, Q. Bao, Q. Zhang, R. Krahne, R. E. Galian, S. D. Stranks, S. Bals, V. Biju, W. A. Tisdale, Y. Yan, R. L. Z. Hoye, L. Polavarapu, *ACS Nano* **2021**, *15*, 10775-10981.
